# Comparative protection of small ruminants against *Mannheimia haemolytica* infection by inactivated bacterin and toxoid vaccines

**DOI:** 10.14202/vetworld.2023.68-75

**Published:** 2023-01-10

**Authors:** Dounia Bkiri, Soufiane Elmejdoub, Zahra Bamouh, Ouafaa Fassi Fihri, Mehdi El-Harrak

**Affiliations:** 1 Department of Research and Development, Multi-chemical Industry, Mohammedia, Morocco; 2 Department of Microbiology, Immunology and Contagious Diseases, Institute of Agronomy and Veterinary Medicine Hassan II, Rabat, Morocco

**Keywords:** efficacy, goats, leukotoxin, *Mannheimia haemolytica*, sheep, vaccination

## Abstract

**Background and Aim:**

*Mannheimia haemolytica* causes respiratory infection and mortality in sheep and goats, similar to the effects in cattle, which causes major economic damage. Regular vaccinations alongside good management practices remain the most efficient tools for controlling this disease. Indeed, vaccines against pasteurellosis are available, but results on their efficacy have varied. Therefore, this study aimed to evaluate the efficacy of three vaccines against mannheimiosis in small ruminants.

**Materials and Methods:**

We evaluated three vaccines developed from a local field isolate based on the inactivated bacterium, its toxoid, and a mixture of bacterin/toxoid, which we then tested on sheep and goats. Selected criteria that were evaluated were safety, antibody response, and protection through a challenge. Post-vaccination monitoring was carried out by enzyme-linked immunosorbent assay. The evaluation was based on antibody responses to vaccination in sheep and goats for both bacteria and leukotoxin. Protection was assessed by clinical and lesion scores after the challenge of vaccinated goats with a pathogenic strain.

**Results:**

The three tested vaccines were completely safe, did not cause any adverse reactions, and induced significant antibody titers in immunized animals. Following *M. haemolytica* challenge, unvaccinated goats showed clinical signs with lesions typical of the disease. Meanwhile, the best protection was obtained with the inactivated combined bacterin/toxoid vaccine.

**Conclusion:**

This study highlighted the effectiveness of adding a bacterial toxoid in the vaccine as a promising solution for preventing mannheimiosis in small ruminants. Because of the worldwide distribution of *M. haemolytica* infection, general prophylaxis based on a combined inactivated vaccine could greatly benefit.

## Introduction

*Mannheimia haemolytica* of the Pasteurellaceae family is the major cause of respiratory diseases in ruminants. It is responsible for direct or indirect economic losses in the livestock industry [1]. *Mannheimia haemolytica* is also associated with outbreaks of severe pneumonia in sheep and goats, as well as septicemia in lambs and mastitis in ewes [2]. Its associated disease is also a major cause of cattle morbidity and mortality [3, 4]. Mannheimiosis is distributed worldwide and occurs in a manner dependent on serotypes in temperate, subtropical, and tropical climates [5, 6]. It is a Gram-negative coccobacillus, aerobic, and non-motile. *Mannheimia haemolytica* occurs naturally as a commensal in the upper respiratory tract microbiota of ruminants. Infection is initiated when the animal’s immune system is compromised by predisposing factors such as stress, transportation, weather, or recurrent viral and mycoplasma diseases [7, 8].

*Mannheimia haemolytica* produces virulence factors consisting of capsular polysaccharides, lipopolysaccharides (LPS), adhesins, outer membrane proteins, iron-binding proteins, secreted enzymes, endotoxin, the ruminant-specific repeats-in-toxin, and exotoxic leukotoxin A (LKT) [9]. Among these, leukotoxin is an immunogenic protein that is pivotal in inducing pneumonia, and responsible for cytotoxic damage of macrophages and leukocytes, in addition to other minor virulence factors. Despite the presence of clinical cases and mortality due to pasteurellosis in small ruminants, limited data on the efficacy of available commercial vaccines have been reported. Vaccination is the best tool to limit the cost of medical treatment for pasteurellosis control [10, 11]; however, vaccines based on inactivated bacteria failed to induce effective protection, and a formulation based on LKT was proven to be more efficient in cattle [12, 13].

This study aimed to develop an efficient vaccine to prevent infection in small ruminants. We tested three vaccine preparations based on LKT toxoid, bacterin, and toxoid-bacterin mixture. The three vaccine formulations were tested for safety and efficacy in goats and sheep, in comparison with unvaccinated control.

## Materials and Methods

### Ethical approval

The study protocol was approved by the Internal Ethic Committee for Animal Experiments (MCI/CEI/0619), and experiments on goats and sheep were performed following the International Guidelines for caring, and handling of experimental animals as described in Chapter 7.8 of the Terrestrial Animal Health Code, and Directive 2010/63/UE of the European Commission [14].

### Study period and location

The study was conducted from January 2021 to December 2021 at Department of Research and Development, Multi-chemical Industry, Mohammedia, Morocco.

### Antigen and vaccine preparations

Preparation of antigens was performed using the Mha serotype 1, a strain of North African origin [6]. The strain was cultured on brain heart infusion (BHI; Solabia, France) agar for 9 h at 37°C, passaged in BHI broth, and incubated for 12 h at 37°C with moderate agitation. Culture in a bioreactor was conducted at 35°C with automatic agitation, and aeration until the end of the logarithmic growth phase reached after 7–9 h of fermentation [15]. After harvesting, the culture was inactivated with 0.5% (v/v) formaldehyde. The bacterial suspension was harvested after centrifugation at 8000× *g* for 30 min at 4°C. To obtain leukotoxin supernatant, the recovered supernatant was treated by ultrafiltration to concentrate the protein [15, 16]. Three vaccines were prepared. The first and second were monovalent (LKT and bacterium-based), while the third was a combined vaccine based on the two antigens (bacterium and LKT). These vaccines were prepared by the adsorption of inactivated antigens with aluminum hydroxide.

### Vaccination

Sixteen sheep and eight goats of local breeds, aged 3–4 months, were acquired from a recognized breeding farm with no history of pasteurellosis infection or vaccination. Before the experiment, animals were allowed to acclimate for 15 days and were monitored daily for general behavior and health status. They were fed a complete and balanced diet and adequate water during this period. Animals were housed in the ABSL2 facility and tested negative for *M. haemolytica* antibodies using a commercial Monoscreen Ab indirect enzyme-linked immunosorbent assay (ELISA) kit (Bio-X Diagnostics, Belgium) [17]. Goats and sheep were divided into four homogeneous groups of four sheep and two goats each. Groups 1–3 were vaccinated with 2 mL by the subcutaneous (SC) route at day 0 and reimmunized on day 28. The first and second groups were vaccinated with 2 mL of inactivated monovalent vaccine based on LKT and Mha, respectively, while the third group received 2 mL of the inactivated combined vaccine (LKT-Mha). Group 4 was an unvaccinated control group. Animals were monitored for 2 weeks for rectal temperature, general health condition, inflammation at the site of inoculation, and appearance of any clinical signs. Serum samples were collected weekly for serological monitoring on days 0, 7, 14, 21, 28, 35, 42, and 2 months in sheep and on days 0, 7, 14, 21, 28, 35, 42, 49, and 56 in goats.

### Serological response to vaccination

The serological response was monitored by ELISA. Blood samples were collected in plain vacuum tubes through jugular venipuncture using an 18G needle, and samples of sera separated from total blood were stored at −20°C until analysis.

### Enzyme-linked immunosorbent assay

Antibodies against *M. haemolytica* cells and against LKT were identified by ELISAs. Lipopolysaccharides of *M. haemolytica*-specific antibodies were assessed using a commercial Monoscreen Ab indirect ELISA kit (Bio-X Diagnostics), in accordance with the manufacturer’s instructions. Briefly, serum samples were added to 96-well plates, coated with LPS of *M. haemolytica*, and incubated for 90 min at 21°C. Wells of the plate were washed with a washing solution, after which 100 μL of the conjugate was added to each well. After incubation for 30 min at 21°C, wells were washed and each supplemented with 100 μL of tetramethylbenzidine (TMB) substrate solution. After 15 min of incubation at 21°C in the dark, the reaction was stopped by adding stop solution, and optical density was measured at 450 nm. Sera were considered negative if the ratio S/P was ≤23% and positive if it was ≥23%.

The leukotoxin antibody response was monitored by ELISA developed in the laboratory. The LKT was suspended in 100 μL of bicarbonate buffer and bound to each well of a Maxisorp plate by incubation overnight at 4°C. The plates were washed with phosphate-buffered saline (PBS) and then goat and sheep-collected sera diluted in 100 μL of blocking buffer were added to the plates. After incubation for 1 h at 37°C, the plates were washed with PBS Tween and incubated with 100 μL of horseradish peroxidase-conjugated anti-immunoglobulin G for an additional 1 h at 37°C. Then, the plates were washed with PBS Tween and TMB substrate was added to each well. After 15 min of incubation at room temperature (21°C±3°C), the reaction was stopped by adding 100 µL of sulfuric acid per well, and the absorbance was read at 450 nm.

### Challenge study

A recent study confirmed the higher sensitivity of goats to *M. haemolytica* than that of sheep [15]. To assess the conferred immunity in this study, we performed experimental infection only on goats. Two weeks after the second injection, the goats along with two unvaccinated control animals were challenged with *M. haemolytica* serotype 1, isolated from an outbreak during winter 2019 in Northern Morocco. Vaccinated and control goats were inoculated with 10^9^ UFC/mL twice at D42 and D43, with 2 mL of the suspension in each nostril, and 5 mL in the distal part of the trachea, using a sterile syringe. Infected goats were observed daily for 2 weeks and blood samples were obtained at D42, D49, and D56. They were observed for clinical signs and rectal temperature and were sampled for antibody detection in blood. A clinical scoring system was applied, which allowed the grading of symptoms and lesions severity. The scale varied from 0 to 4, depending on symptom severity (Table-1), and from 0 to 6, depending on the recognizable lesions of the disease (Table-2).

**Table-1:** Scoring of recorded clinical signs of challenged goats.

Clinical signs	Score
General behavior	
Normal	0
Inactive	2
Very inactive	4
Fever	
Normal	0
39°C–40°C ≤2 days	2
39°C–40°C ≥2 days	3
>40°C	4
Food uptake	
Normal	0
Loss of appetite	2
Anorexia	4
Local inflammation	
None	0
<2 cm	1
>2 cm	2
Cough	
Normal	0
<2 days	1
2–4 days	2
>days	4
Respiratory rate	
Normal	0
Tachypnea	2
Dyspnea	4
Nasal secretions	
Absence	0
Presence	2

**Table-2:** Scoring of recorded lesions of challenged goats.

Lesional signs	Score
Atelectasis	
Normal	0
≤25	2
25%–50%	4
50%–75%	6
Congestion	
Normal	0
≤25	2
25%–50%	4
50%–100%	6
Trachea	
Normal	0
Presence of foam	2
Congestion	4
Lung nodes	
Normal	0
Slightly swell	2
Moderately swell	4
Very swell	6
Consolidation	
Normal	0
≤25	2
25%–50%	4
50%–100%	6
Hepatization	
Normal	0
≤25	2
25%–50%	4
50%–100%	6

## Results

### Post-vaccination monitoring in sheep

**Figure-1:**
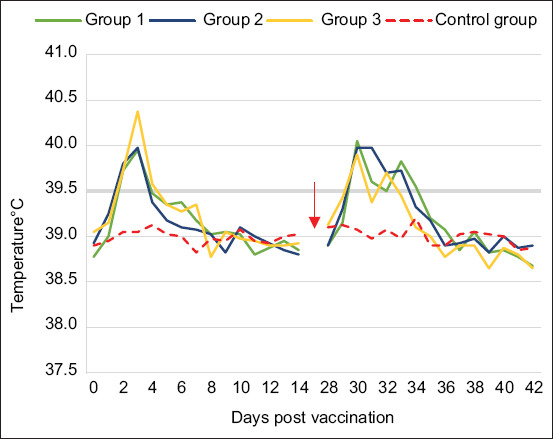
Average body temperature monitoring of vaccinated versus unvaccinated sheep: G1 vaccinated with toxoid vaccine, G2 vaccinated with inactivated bacteria Mha vaccine, G3 vaccinated with combined vaccine (LKT/Mha), and Control group. The red arrow indicates the time period in which sheep from G1, G2, and G3 were received the second injection. LKT=Leukotoxin A.

An increase in body temperature was noted between D2 and D4 post-vaccination in the three vaccinated groups. G3 vaccinated with inactivated toxoid-bacterin was the only one with a peak temperature exceeding 40.3°C, at D3 post-vaccination. The average temperature for each group is reported in Figure-1. A transient local reaction at the injection site was observed in some animals, which disappeared within a few days.

**Figure-2:**
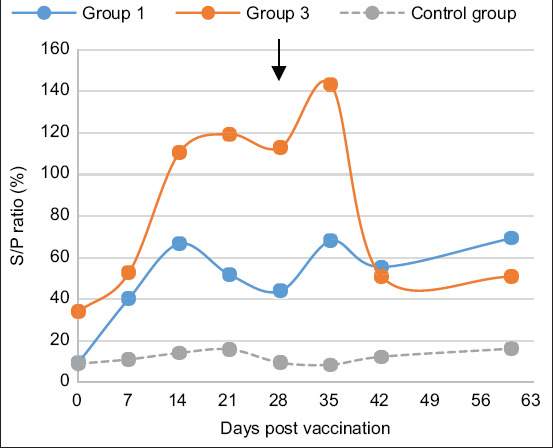
Average LKT antibody response in sheep after vaccination. Groups 1 and 3, four sheep each, were vaccinated with toxoid vaccine and combined vaccine (LKT/Mha), respectively. The antibody kinetic was performed by ELISA antibodies against leukotoxin of Mha. The arrow corresponds to the booster. ELISA=Enzyme-linked immunosorbent assay, LKT=Leukotoxin A.

**Figure-3:**
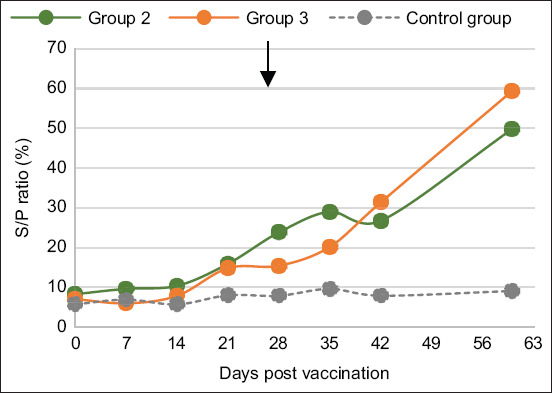
Average whole-bacteria antibody response in sheep after vaccination. Groups 2 and 3, four sheep each, were vaccinated with inactivated bacteria Mha vaccine and combined vaccine (LKT/Mha), respectively. The antibody kinetic was performed by a commercial Monoscreen Ab indirect ELISA kit. The s/p ratio ≥23% was considered positive. The arrow corresponds to the booster. ELISA=Enzyme-linked immunosorbent assay, LKT=Leukotoxin A.

Vaccination of sheep in G1 and G3 groups generated significant amounts of anti-LKT antibodies (Figure-2). A difference between the two groups was noted, in that titers of LKT antibodies started to increase on day 7 post-vaccination for both groups, but LKT Ab response with the combined vaccine was higher than with the monovalent LKT vaccine, and peaked at 143% at D35 following the second injection, compared with the level in G1 (68% at D35). Animals developed antibody response from D7 post-vaccination for sheep and D14 post-vaccination for goats. Regarding the serological response to Mha (Figure-3), the antibody titers after the vaccination of sheep were slightly higher in the G2 group injected with the killed bacterin than in the G3 group vaccinated by the combined Mha and LKT vaccine. The seroconversion for both groups occurred later than seroconversion to LKT. Remarkably, the G3 group exhibited seroconversion only after the second injection and showed a peak at D60 (Figure-3). The control group remained negative throughout the trial of the three tested vaccines.

### Vaccination monitoring in goats

**Figure-4:**
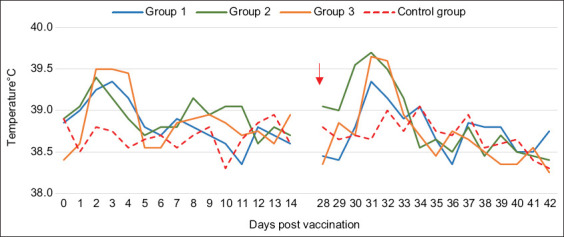
Average body temperature monitoring of vaccinated versus unvaccinated goats: G1 vaccinated with toxoid vaccine, G2 vaccinated with inactivated bacteria Mha vaccine, G3 vaccinated with combined vaccine (LKT/Mha), and Control group. The red arrow indicates the time period in which goats from G1, G2, and G3 were received the second injection. LKT=Leukotoxin A.

A slight increase in body temperature was noted between D1 and D5 post-vaccination in goats of the three groups. They remained healthy without any adverse reactions at the site of the injection, with an average body temperature ranging from 38.4°C to 39.7°C. The temperature was normal in the unvaccinated control group (Figure-4).

### Protection against challenge

**Figure-5:**
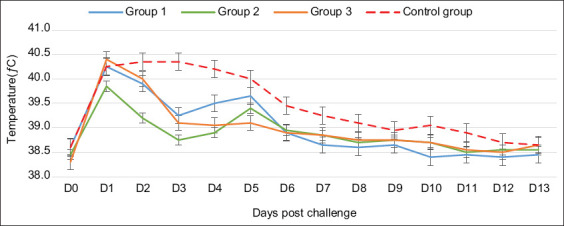
Average body temperature monitoring of vaccinated and challenged goats: G1 vaccinated with toxoid vaccine, G2 vaccinated with inactivated bacteria Mha vaccine, G3 vaccinated with combined vaccine (LKT/Mha), and Control group. LKT=Leukotoxin A.

**Figure-6:**
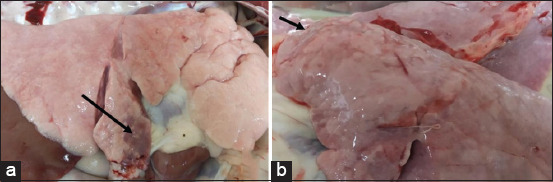
Figure of challenged goats after inoculation of Mha strain and necropsy, showing the affected portions of the lungs: (a) Well-demarcated red hepatization areas affecting the ventral lung lobes (black arrows) and (b) multifocal consolidated areas affecting cranioventral lung lobe (black arrow).

Two weeks after the second injection, the four groups of vaccinated and unvaccinated goats were challenged, as described previously. Goats in the control group showed an elevated body temperature starting the 1^st^ day after the challenge, which remained high until D5 post-injection, peaking at D2 and D3 above 40°C (Figure-5). The unvaccinated goats showed clinical symptoms of the disease and presented a higher score (40) than the vaccinated groups (9 for G1, 11 for G2, and 7 for G3 as the lowest score) (Table-3). At necropsy, the following conditions were observed in the control group: Atelectasis, congestion, consolidation, and hepatization (Figure-6). The other three vaccinated groups presented only mild hyperthermia after the challenge and moderate lesions of respiratory disease.

**Table-3:** Clinical and lesion scores in vaccinated and unvaccinated goats after challenge.

Group	Clinical score	Lesion score	Total
Unvaccinated group	10	30	40
Group 1 (LKT vaccine)	3	6	9
Group 2 (Mha vaccine)	3	8	11
Group 3 (combined vaccine)	2	5	7

### Serological response after vaccination and challenge

**Figure-7:**
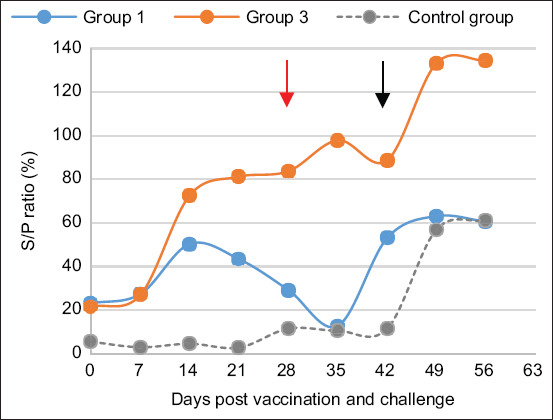
Average LKT antibody response in goats after vaccination, booster, and challenge by Mha. Groups 1 and 3, two goats each, were vaccinated with toxoid vaccine and combined vaccine (LKT/Mha), respectively. The antibody kinetic was performed by ELISA for antibodies against leukotoxin of Mha. The red arrow corresponds to the booster and the black arrow corresponds to the challenge. ELISA=Enzyme-linked immunosorbent assay, LKT=Leukotoxin A.

Goats vaccinated with LKT vaccine (G1) showed seroconversion after primary vaccination starting on day 14 post-vaccination, which decreased on day 35 post-vaccination, while a slight increase in LKT antibodies was noted after the challenge. Remarkably, serological response in the combined vaccine (G3) group was higher than in the monovalent (G1) group (Figure-7).

**Figure-8:**
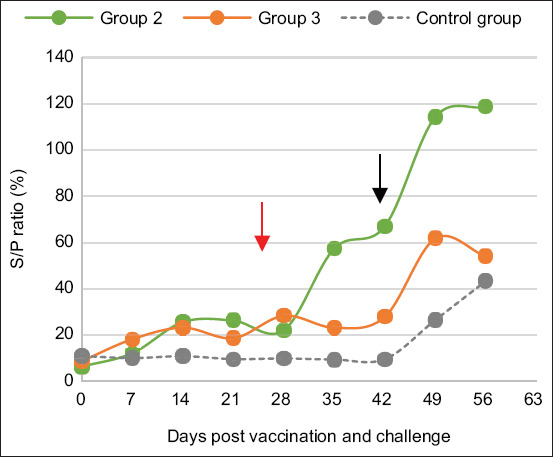
Average whole-bacteria antibody response in goats after vaccination, booster, and challenge by Mha. Groups 2 and 3, two goats each, were vaccinated with inactivated bacteria Mha vaccine and combined vaccine (LKT/Mha), respectively. The antibody kinetic was performed by a commercial Monoscreen Ab indirect ELISA kit. The S/P ratio ≥ 23% was considered positive. The red arrow corresponds to the booster and the black arrow corresponds to the challenge. LKT=Leukotoxin A.

Seroconversion to bacterial cells of the G2 and G3 groups was similar after vaccination, but the booster effect was more noted in G2 (bacterially vaccinated), with a peak after the challenge. The G4 group of unvaccinated goats exhibited seroconversion only after the challenge, which peaked at D49 (Figure-8).

## Discussion

The most effective tool to limit financial losses due to mannheimiosis in ruminants is to conduct vaccination in endemic areas, using effective vaccines. Sustainable vaccination campaigns associated with appropriate breed management are the key preventive measures to minimize the impact of the disease [18]. Vaccination with *M. haemolytica* bacterins failed to induce effective protection, probably because this bacterium produces some virulence factors involved in clinical disease and mortality [19]. The Mha leukotoxin is known to be cytotoxic to ruminant leukocytes and has been the subject of studies on its virulence and immunogenicity in cattle [15, 20]. The presence of this protein in the vaccine components may contribute to an increase in its efficacy.

Unlike in cattle, few vaccines have been developed and used to prevent mannheimiosis in sheep and goats, despite the huge impact of this disease in those species [17, 21]. To the best of our knowledge, no information has been reported on small ruminants’ regular vaccination against *M. haemolytica* or the vaccine’s composition and efficacy in those species. In this study, we evaluated the efficacy of three vaccine preparations based on toxoid, inactivated bacteria, or both. Indeed, evaluation of the protective effect was based on antibody responses in sheep and goats, and resistance to challenge in goats as the species most sensitive to the infection [22]. The objective was to determine whether the support of the infection and immunity is more related to bacterial cells or to excreted leukotoxin in small ruminants, as was documented in cattle. Inactivated antigens were adsorbed with aluminum hydroxide as an adjuvant under identical conditions. The choice of adjuvant was based on results of the previous study, which reported its efficacy and ability to enhance immune responses after vaccination [23].

In vaccinated sheep and goats with the monovalent LKT and combined LKT/Mha vaccines, the production of LKT antibodies was induced, as detected by ELISA, in accordance with the previous reports in cattle [24–26]. Antibody titers obtained after vaccination significantly higher than those with the vaccine based on toxoid alone. It appears that the presence of bacteria in the vaccine boosted the antibody response to LKT, likely due to residual intracellular LKT or the adjuvant effect of bacterial cells, as observed by Ayalew *et al*. [27] in mice.

Regarding the serological response to *M. haemolytica*, vaccinated sheep and goats reacted later to the bacteria (D35 after booster) than to its LKT (D7/14 after primary vaccination). This was probably related to the immunodominance of the LKT antigen compared with the whole bacterial cells. After the booster, sheep vaccinated with Mha and Mha/LKT responded similarly, while goats vaccinated with Mha presented a more intense response to bacterial cells than those vaccinated with the combination. This result is in accordance with Srinand *et al*. [26], who recommended that vaccination in cattle with a combined vaccine requires a booster, and claimed that the highest response was seen only at D14 following the booster. Shewen *et al*. [28] also reported that the use of two vaccine doses protects cattle against pneumonia at a rate of 60%–70% [28]. Vaccines administered at a single dose may not be effective unlike what has been recommended by some authors who judge that a booster effect occurs in the field by natural exposure [26].

In this study, the experimental infection showed full protection of vaccinated animals following the challenge, in contrast to the findings in unvaccinated controls. The combined vaccine was proven to be efficient in conferring resistance to *M. haemolytica*, since animals in the G3 group presented lower clinical and lesion scores than the other two groups. The vaccine based on toxoid and bacterin could elicit an immunological response and protect against infection, as reported by Confer *et al*. [12]. Unvaccinated challenged goats showed clinical signs that peaked at 24–48 h after the challenge, proving that the used dose (10^9^ UFC) was sufficient to reproduce typical lesions in this species, despite the absence of predisposing factors such as stress or viral coinfection. The poor sensitivity of goats to Mha experimental infection as reported by several authors [15, 29] may explain the weak difference in symptoms and lesion scores among the three vaccinated groups. Interestingly, these results confirmed that LKT is a key component of the vaccine, which was also confirmed in the previous studies [30, 31], and provides evidence that an inactivated vaccine based on toxoid and bacterin is efficient, can induce high antibody responses, and provides protection against challenge. This observation has also been highlighted by Confer *et al*. [12] in a review of Mha vaccination. In that study, experimental infection showed full protection of vaccinated goats following a challenge carried out 42 days after vaccination.

## Conclusion

*Mannheimia haemolytica* infection in small ruminants, similar to cattle, causes typical respiratory symptoms and lesions, as shown in the experimental infection of goats in this study. Furthermore, the inactivated combined Mha-LKT vaccine provides a beneficial solution to protect livestock against respiratory diseases due to *M. haemolytica*. This vaccine is completely innocuous and efficient for use in endemic regions.

## Authors’ Contributions

DB: Performed the experiments, analyzed the data, and drafted the manuscript. SE: Performed serological analysis. ZB and DB: Carried out the experiment on goat and sheep. OFF: Participated in the design and follow-up of the study. ME: Participated in the design of the study, manuscript drafting, data analysis, and interpretation. All authors have read and approved the final manuscript.
